# A spatiotemporal gating hypothesis for psilocybin plasticity: reconciling the 5-HT₂A-TrkB mechanistic paradox

**DOI:** 10.1038/s41421-026-00906-4

**Published:** 2026-05-29

**Authors:** Gang Pei

**Affiliations:** 1 State Key Laboratory of Cell Biology, Shanghai Institute of Biochemistry and Cell Biology, Center for Excellence in Molecular Cell Science, Chinese Academy of Sciences, Shanghai, China; 2 Shanghai Key Laboratory of Signaling and Disease Research, School of Life Sciences and Technology, Tongji University, Shanghai, China

Psilocybin has emerged as a transformative, rapid-acting, and long-lasting therapeutic for treatment-resistant depression, with its efficacy rooted in the induction of sustained structural and functional remodeling of mood-regulating neural circuits^1,2^. However, the field faces a fundamental mechanistic paradox that defies linear signaling models: psilocin, the bioactive metabolite of psilocybin, directly binds to and allosterically potentiates TrkB receptors — the primary transducers of BDNF-mediated neurotrophic and plastic effects^3^ — yet deletion or pharmacological blockade of 5-HT₂A receptors completely ablates psilocybin-induced dendritic spine formation, circuit rewiring, and long-term antidepressant behavior^1,4^. This strict dependency on 5-HT₂A, despite direct TrkB engagement, raises a critical question: what is the functional relationship between these two receptors in mediating psilocybin’s plastic effects?

Here, we propose a spatiotemporal gating hypothesis — synthesized from published genetic, cellular, and behavioral data — that resolves this paradox by defining 5-HT₂A and TrkB not as redundant or sequential components of a single pathway, but as a coordinated system in which 5-HT₂A acts as a mandatory spatiotemporal gate and temporal primer, and TrkB serves as the structural executor of synaptic rewiring (Fig. 1). For psilocybin to induce functional neuroplasticity, TrkB activation must occur within the 5-HT₂A-defined spatial substrate and temporal window; outside this context, direct TrkB engagement remains functionally silent.

**Fig. 1:**
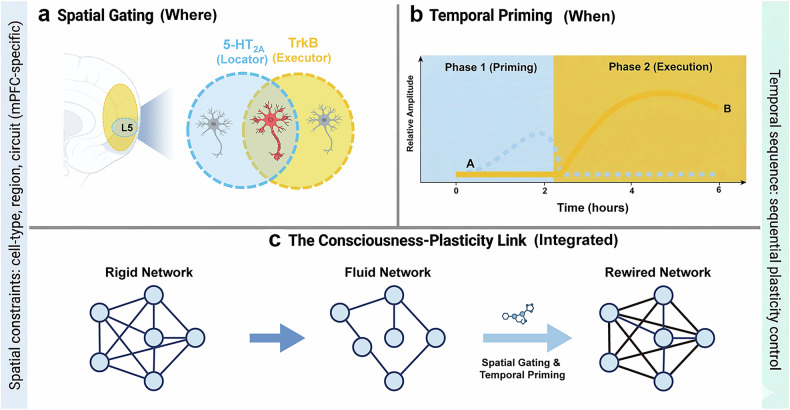
A spatiotemporal gating hypothesis for psilocybin-induced neural plasticity. **a** Spatial gating: 5-HT₂A receptors act as a “spatial spotlight” selectively enriched in the apical dendrites of layer V pyramidal tract (PT) neurons in the medial prefrontal cortex (mPFC). TrkB receptors are ubiquitously expressed, but their signaling drives functional synaptic remodeling only within the 5-HT₂A-enriched spatial substrates^4^. **b** Temporal priming: psilocybin acts in an irreversible two-phase sequence. Phase 1 (priming): 5-HT₂A activation triggers calcium transients and mTOR signaling^5^, opening a transient permissive window for plasticity. Phase 2 (execution): psilocin-mediated TrkB potentiation stabilizes nascent dendritic spines only if TrkB engagement occurs within the Phase 1 window^1,3^. **c** Functional coordination: the convergence of 5-HT₂A-mediated spatial gating and temporal priming enables TrkB to drive sustained synaptic remodeling and circuit rewiring in the mPFC, the cellular and anatomical basis of psilocybin’s long-term antidepressant effects^1,2^. Without this spatiotemporal coordination, direct TrkB activation is insufficient for functional plasticity.

## Spatial gating: restricting plasticity to specialized cellular substrates

Psychedelic-induced plasticity is not a diffuse, global process but is constrained to specific cellular and anatomical substrates that underpin higher-order emotional and cognitive regulation — an observation that forms the basis of spatial gating of our hypothesis. 5-HT₂A receptors are densely and selectively concentrated in the apical dendrites of layer V pyramidal tract (PT) neurons in the medial prefrontal cortex (mPFC)^4^, a cell population critical for modulating mood and depressive behavior. These receptors act as a spatially permissive gate: although TrkB receptors are ubiquitously expressed across multiple neuronal and non-neuronal cell types, their capacity to drive productive, functional synaptic reorganization is functionally “unlocked” only within these 5-HT₂A-enriched layer V PT neurons (Fig. 1a).

This spatial constraint is directly supported by genetic evidence: conditional deletion of 5-HT₂A specifically in mPFC layer V PT neurons abolishes psilocybin-induced dendritic spine growth and long-term antidepressant effects in vivo, while TrkB activation in other cell populations fails to compensate for this loss^4^. Without this 5-HT₂A-mediated spatial gate, TrkB signaling represents a signal without a functional context — unable to target the specific neural circuits required for reversing maladaptive depressive plasticity.

## Temporal priming: a transient permissive window for TrkB execution

In addition to spatial constraints, psilocybin’s plastic effects follow a nonreversible, strict temporal sequence — the second core of our spatiotemporal gating hypothesis — in which 5-HT₂A activation must precede and prime the neuron for TrkB-mediated structural change (Fig. 1b). Within minutes of psilocybin administration, 5-HT₂A receptor activation triggers Gq-mediated calcium transients and downstream activation of the mTOR signaling pathway^1,5^, a master regulator of synaptic protein synthesis and structural plasticity. This 5-HT₂A-dependent priming phase mobilizes the molecular machinery required for the formation and stabilization of nascent dendritic spines, effectively opening a transient permissive window for plastic change.

Crucially, psilocin-mediated TrkB potentiation drives the maturation and long-term stabilization of these new synapses only if it occurs within this 5-HT₂A-dependent window^1,3^. If 5-HT₂A signaling is absent, this temporal window never opens; even direct TrkB activation cannot overcome the absence of pre-assembled plastic machinery, and functional synaptic remodeling does not occur. In this model, 5-HT₂A provides the molecular “permission” for plasticity, while TrkB executes the structural “construction” of new synapses; both steps are mandatory, and their sequence is non-negotiable.

## Functional implications for rapid-acting antidepressant pharmacology

This spatiotemporal gating hypothesis provides a unifying framework for understanding the receptor coordination that underpins psilocybin’s uniquely rapid and sustained antidepressant effects and has broader implications for the pharmacology of rapid-acting antidepressants. A key insight is that direct activation of plasticity-related signaling pathways (e.g., TrkB) is insufficient for therapeutic efficacy — the signal must be targeted to the correct cellular substrate and timed to coincide with a permissive molecular state, both of which are controlled by a dedicated gatekeeper receptor (here, 5-HT₂A).

This model also explains why non-hallucinogenic TrkB agonists failed to replicate psilocybin’s long-term antidepressant effects in preclinical and early clinical studies: these compounds lack the 5-HT₂A-mediated spatiotemporal gating required to target TrkB activation to mPFC layer V PT neurons and to align with the transient permissive window during which plastic change is possible. For next-generation psychedelic-based therapeutics, this hypothesis identifies a critical design principle: effective compounds must either co-opt the endogenous 5-HT₂A spatiotemporal gate or mimic its ability to restrict and prime TrkB-mediated plasticity within the relevant cellular and temporal substrates.

In summary, the 5-HT₂A-TrkB paradox in psilocybin research is resolved not by linear signaling but by a spatiotemporal gating system in which 5-HT₂A and TrkB act in coordination to mediate functional neuroplasticity. 5-HT₂A defines the “where” and “when” of plasticity, whereas TrkB defines the “what”; without the former, the latter cannot produce meaningful therapeutic change. This hypothesis is rooted in the synthesis of existing experimental evidence and provides a testable framework for future studies of psychedelic pharmacology and the design of novel rapid-acting antidepressants.
